# Change in BMI Accurately Predicted by Social Exposure to Acquaintances

**DOI:** 10.1371/journal.pone.0079238

**Published:** 2013-11-20

**Authors:** Rahman O. Oloritun, Taha B. M. J. Ouarda, Sai Moturu, Anmol Madan, Alex (Sandy) Pentland, Inas Khayal

**Affiliations:** 1 Institute Center for Smart and Sustainable Systems (iSMART) Masdar Institute of Science and Technology, Abu Dhabi, UAE; 2 Institute Center for Water and Environment (iWATER), Masdar Institute of Science and Technology, Abu Dhabi, UAE; 3 The Media Lab, Massachusetts Institute of Technology, Cambridge, Massachusetts, United States of America; University College London, United Kingdom

## Abstract

Research has mostly focused on obesity and not on processes of BMI change more generally, although these may be key factors that lead to obesity. Studies have suggested that obesity is affected by social ties. However these studies used survey based data collection techniques that may be biased toward select only close friends and relatives. In this study, mobile phone sensing techniques were used to routinely capture social interaction data in an undergraduate dorm. By automating the capture of social interaction data, the limitations of self-reported social exposure data are avoided. This study attempts to understand and develop a model that best describes the change in BMI using social interaction data.

We evaluated a cohort of 42 college students in a co-located university dorm, automatically captured via mobile phones and survey based health-related information. We determined the most predictive variables for change in BMI using the least absolute shrinkage and selection operator (LASSO) method. The selected variables, with gender, healthy diet category, and ability to manage stress, were used to build multiple linear regression models that estimate the effect of exposure and individual factors on change in BMI. We identified the best model using Akaike Information Criterion (AIC) and R^2^.

This study found a model that explains 68% (p<0.0001) of the variation in change in BMI. The model combined social interaction data, especially from acquaintances, and personal health-related information to explain change in BMI.

This is the first study taking into account both interactions with different levels of social interaction and personal health-related information. Social interactions with acquaintances accounted for more than half the variation in change in BMI. This suggests the importance of not only individual health information but also the significance of social interactions with people we are exposed to, even people we may not consider as close friends.

## Introduction

Reports from the World Health Organization (WHO) indicate that obesity has reached epidemic proportions globally, with over a billion people said to be overweight [1]. However, research has mostly focused on obesity and not on processes that change BMI more generally, although these may be key factors that lead to obesity. Recent studies in public health have suggested that obesity and other health related behaviors are impacted by social networks and that social support is a key factor in an individual's health and well-being [2]–[4]. People are part of social networks and can be influenced by appearances and behaviors of other people around them [5]–[7]. This suggests that weight change in one person can influence weight change of others.

Furthermore, longitudinal studies from the Framingham Heart study suggest that health related behavior from obesity [8] to happiness [9] can spread through social ties.

These studies depend on self-reported data collected over prolonged periods of time, and may be subject to inaccuracies [10]. For example, the social network ties in the original Christakis-Fowler work were generated from respondent surveys that were updated only once every three years and only requested respondents to mention a friend, spouse and parents.

Gathering data on human interactions utilizing routine or generally accepted techniques, such as surveys, interviews, is constrained in spatial and time scales by technical difficulties and cost [5]. Lately, digital traces of human actions are becoming available and are enabling modeling and analysis of massive amounts of data on human behavior. Monitoring human behavior, choices and outcomes in an assortment of settings has become feasible at different spatial and time scales. Behavior of humans ranging from mobility of individuals within a dormitory [7], city [11] and between cities [12] in countries [13], and globally [5] can be automatically captured using ubiquitous sensor devices, such as cell phones. These devices have also made it feasible to study patterns of mobility [14], [15].

In this study, we used mobile phone sensing techniques to routinely capture social interaction data in an undergraduate dorm. By automating the capture of social interaction data, we avoided the limitations of self-reported social exposure data. To our knowledge, this is the first study that attempts to understand the effect of exposure on the change in BMI in a sensor delineated face-to-face network of individuals.

## Methods

### Study Population and Data Collection

This study was performed in a real-world setting of an American university undergraduate dormitory during the spring of 2009. The analysis included 42 subjects composed of freshmen, sophomores, juniors, seniors and graduate resident tutors responsible for each floor in the residence. The subjects ranged from 20–30 years of age with a median age of 22 years. There were 21 females and 21 males.

All subjects in the study were given ‘socially-aware’ mobile phones that used Bluetooth sensors/transceivers to detect other proximate phones in the study. Once a phone detects another phone in proximity, it captures the other phone's identifier and records the length of time the phones were in proximity. A detailed description of the data collection platform and the technologies used is available elsewhere [10], [16], [17]. The interaction data was aggregated for different periods of time including: the total time, the entire period of the study; weekday, from midnight Monday to 9pm on Friday; and weekend, from 9pm on Friday to midnight Monday.

Subjects completed a monthly health-related survey, which included information about dietary habits, physical exercise, weight, height, and stress level for the months of March, April and May 2009. Subjects also indicated their close friendships (binary responses). The dataset is included at the following URL: http://realitycommons.media.mit.edu/SocialEvolution.zip.

### Ethics Statement

The study was approved by the Massachusetts Institute of Technology Institutional Review Board (IRB) called the Committee On the Use of Humans as Experimental Subjects (COUHES) and conducted under strict protocol guidelines. Participants provided their written informed consent to participate in this study.

### Data Aggregation and Variables

**Figure 1:**
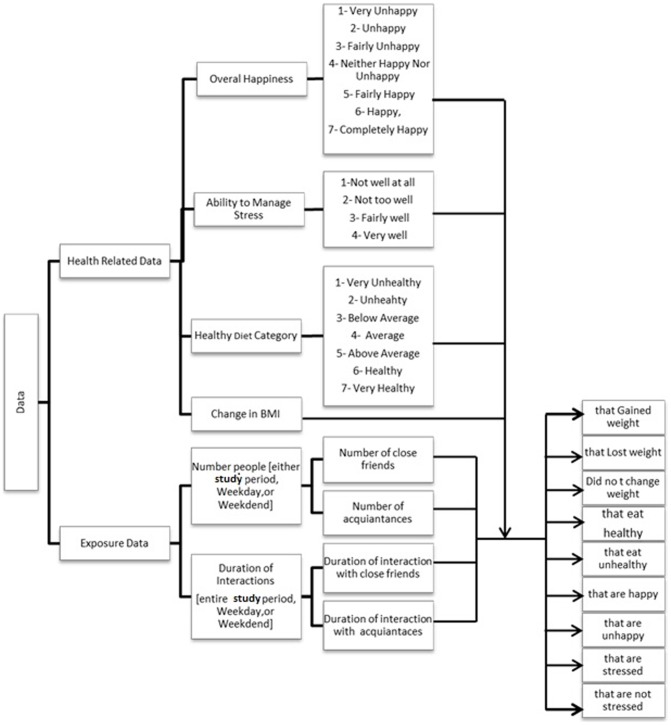
The hierarchy of the data consisting of two main groups, the exposure and health-related information.

The hierarchy of the data used in this study is shown in figure 1. The data consist of two classes of data, social exposure and health-related information.

### Change in BMI

**Figure 2:**
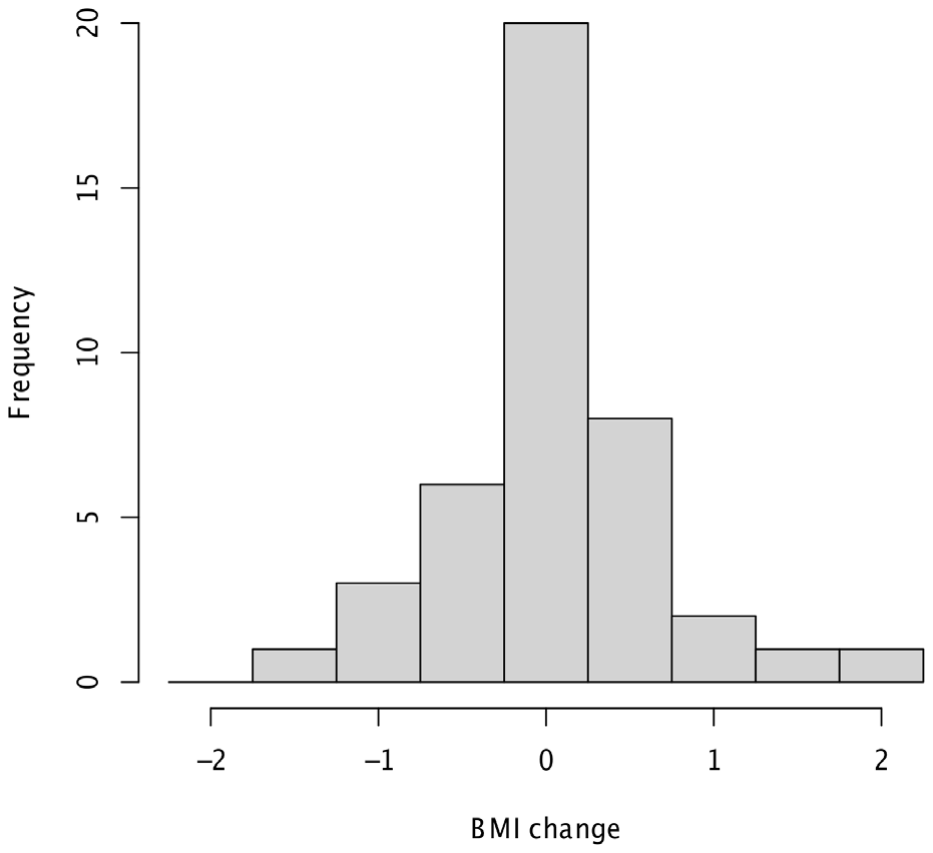
Histogram showing the change in BMI (difference of the BMI measured at the beginning and end of the study) between March and May 2009.

The change in BMI is the difference of the BMI measured at the beginning and end of the study and represents the dependent variable. We identify subjects who gained weight as individuals with weight change above the threshold value of 1% change in weight and subjects who lost weight as individuals with weight change below the threshold value of −1% change in weight. A histogram showing change in BMI between March to May 2009 is shown in figure 2.

### Health-Related Variables

The healthy diet category was captured on the Likert scale of 1–7 (1-very unhealthy, 2-unhealthy, 3-below average, 4-average, 5-above average, 6-healthy, and 7-very healthy). Happiness was captured on the scale of 1–7 (1-very unhappy, 2-unhappy, 3-fairly unhappy, 4-neither happy nor unhappy, 5-fairly happy, 6-happy and 7-completely happy). Ability to manage stress was captured on a 1–4 scale (1-Not well at all, 2-Not too well, 3-Fairly well, 4-Very well).

Using the healthy diet category scale for each subject, we obtained the duration of interaction with and number of people classified as unhealthy (subjects with responses less than or equal to 3 on the healthy diet category scale) and healthy (subjects with responses greater than 3 on the healthy diet category scale) that interacted with each subject. With the overall happiness for each subject, we identified the duration of interaction with and number of subjects who are unhappy (response to overall happiness is less than or equal to 4) and happy (response to overall happiness is greater than 4) that interacted with each subject. We identified the duration of interactions with and number of subjects who are stressed (response to ability to manage stress less than or equal 2) and are not stressed (response to ability to manage stress is greater than 2) that interacted with each subject.

### Measures of Social Exposure

We identified two possible measures of exposure from the interaction information capture. These exposure measures are, together with personal choices and attributes, the independent variables for two distinct groups of multivariate exposure models, respectively. These exposure measures are (1) Scored duration of interaction and (2) Number of subjects exposed to.

The first measure is scored duration of interaction which is based on the intuition that subjects interact in diverse patterns with varying duration levels. Therefore, it is imperative that duration of interactions with others is normalized across subjects. To do so, we implemented a function that maps the durations of interactions for each subject that occur in the first, second, third and fourth quartile to values 1, 2, 3, and 4 respectively. This quartile scoring approach retains the magnitude of durations of interaction as a factor in understanding change in BMI. Scored duration of interaction comprises variables that describe total score of the duration of interaction with both close friends and acquaintances. We considered interactions with close friends and acquaintances that gained weight, lost weight, did not change weight, eat healthy, eat unhealthy, are happy, are unhappy, are stressed and are not stressed. These exposure attributes are the sums of the scored durations of interaction by a subject with people that have a specific health-related attribute (e.g. eat healthy, happy, stressed) or outcome such as gain in weight or loss of weight.

The second measure is the number of subjects exposed to which consists of variables such as the numbers of close friends or numbers of acquaintances who gained weight, lost weight, did not change weight, eat healthy, eat unhealthy, are happy, are unhappy, are stressed and are not stressed. These exposure attributes are numbers of people with whom a subject interacts, who have specific health-related or weight change outcomes. All variables were available and included for each participant.

### Data Analysis

We approached the statistical analysis of the data in two steps. The first step is variables selection, which searches for the exposure related parameters that can explain change in BMI. To implement variables selection, we used least absolute shrinkage and selection, LASSO [18], to find variables that best explain the change in BMI using exposure to a variety of health behaviors at different periods in time, (entire period of study, weekday and weekend). LASSO is a powerful penalized regression method used in predictor selection. It allows the analysis to properly deal with collinearity problems for more accurate and clear models while avoiding the complex formulations associated to stepwise models.

We took Mallows C_p_ [19] as explanatory variables selection criterion [20]. The selected parameters were put together with gender, healthy diet category and stress management ability of subjects to develop multivariate linear regression models that explain the change in BMI of the participating subjects. The models were divided into two groups based on type of exposure. Each group consists of exposure models for data aggregated over the entire period of study, weekdays and weekends respectively. We identified the best model using R^2^ and Akaike Information Criterion (AIC). AIC was proposed by Akaike [21] as an information criterion for model selection employing the relationship between the mean value of the logarithms of the likelihood and Kullback-Leibler information. All the linear models were checked to ensure they do not violate the assumptions of ordinary least squares regression (OLS) [22].

## Results

An optimal model that explains 68% (p<0.00001) of the variation in change in BMI was obtained. The model consists of statistically significant exposure terms such as “duration of interaction with acquaintances that gained weight”, “duration of interaction with acquaintances that are not stressed” and “duration of interaction with close friends who are not stressed”. The model also includes significant parameters representing individuals' personal choices of level 4 to level 6 of the healthy diet category and level 3 of the ability to manage stress. The resulting linear regression models are presented in Tables 1 and 2 for the exposure models based on duration of interactions and number of subjects exposed to, respectively.

**Table 1:** A multivariate linear regression model based on the scored duration of interaction with people with a specific health-related behavior or outcome.

	Total Estimate (S.E.)	Weekday Estimate (S.E.)	Weekend Estimate (S.E.)
(Intercept)	1.67* (0.66)	0.94 (0.6)	0.96 (0.59)
Duration of interactions with:			
Close friends who are not stressed	−0.05*** ( 0.01)	−0.04** (0.01)	−0.04** (0.01)
Acquaintances who gained weight	−0.14*** ( 0.03)	−0.11*** ( 0.03)	−0.09** (0.03)
Acquaintances who are not stressed	0.03*** ( 0.01)	0.02** (0.01)	0.03*** (0.01)
Acquaintances who eat unhealthy		0.01 (0.03)	−0.04 (0.03)
Healthy Diet Category (category 2 which is the least from the responses is the reference value)			
3 -Below Average	−0.26 (0.27)	−0.12 (0.29)	−0.44 (0.34)
4 -Average	−0.46* (0.21)	−0.42 (0.23)	−0.43 (0.27)
5 –Above average	−0.53* (0.23)	−0.46 (0.28)	−0.54 (0.31)
6 -Healthy	−1.77*** ( 0.48)	−1.48** (0.53)	−1.64** (0.6)
Ability to manage stress (level 2 which is the least from the responses is the reference value			
3 -Fairly well	0.7*** (0.18)	0.66** (0.19)	0.75** (0.21)
4 -Very well	0.41 (0.24)	0.49 (0.25)	0.73** (0.26)
Gender (Female is the reference gender)			
Male	−0.01 (0.15)	0.03 (0.16)	−0.18 (0.18)
N	42	42	42
RMSE	0.35	0.36	0.41
RSS	0.41	0.44	0.49
R^2^	0.68	0.65	0.57
AdjustedR^2^	0.58	0.52	0.41
p	<0.0001	<0.001	<0.01
AIC	55.61	61.51	70.42

* = p≤0.05,

** = p≤0.01,

*** = p≤0.001.

**Table 2:** A multivariate linear regression model based on number of subjects with specific health-related behaviors or outcomes.

	Total Estimate (S.E.)	Weekday Estimate (S.E.)
(Intercept)	0.72 (1.54)	0.97 (1.42)
Number of:		
Close friends who eat healthy	−0.1 (0.05)	−0.1 (0.05)
Acquaintances who gained weight	−0.38** (0.11)	−0.36** (0.11)
Acquaintances who are not stressed	0.05 (0.04)	0.06 (0.04)
Acquaintances who eat healthy	0.07 (0.1)	0.04 ( 0.1)
Healthy Diet Category (category 2 which is the least from the responses is the reference value)		
3- Below Average	−0.09 (0.33)	−0.16 (0.33)
4 -Average	−0.33 (0.29)	−0.48* (0.28)
5-Above Average	−0.39 (0.35)	−0.48 (0.34)
6 -Healthy	−1.7** (0.6)	−1.66* (0.62)
Ability to manage stress (level 2 which is the least from the responses is the reference value		
3 -Fairly well	0.82*** ( 0.22)	0.73** (0.22)
4-Very well	0.74* (0.27)	0.72* (0.28)
Gender (Female is the reference gender)		
Male	−0.03 (0.18)	−0.06 (0.19)
N	42	42
RMSE	0.42	0.42
RSS	0.49	0.5
R^2^	0.56	0.54
AdjustedR^2^	0.39	0.38
p	<0.01	<0.01
AIC	71.42	72.52

* = p≤0.05,

** = p≤0.01,

*** = p≤0.001.

Furthermore, when individual choices and attributes (diet, stress and gender) were taken out of the optimal model, social exposure alone explains approximately 38% (p<0.001) of the variation in change in BMI, which is more than half of the 68% of the variation in change in BMI explained by the combination of exposure and personal choices. The personal attributes (gender) and health related choices, healthy diet category and ability to manage stress, only explain approximately 26% (p = 0.13) of change in BMI.

The optimal model is based on parameters aggregated for the entire period of the study. A model based on weekday exposure only and a model based on weekend exposure explain approximately 65% (p<0.0001) and 57% (p<0.01) of the variation in change in BMI, respectively with independent variables similar to those in the optimal model (see third and fourth column in Table 1).

However, when we calculate exposure as the number of subjects rather than the duration of interactions, we find a model that explains 56% (p<0.01) of the variation in change in BMI. The significant exposure parameters in this model include the “number of acquaintances that gained weight”, diet and stress (see Table 2).

The model based on weekday exposure (number of subjects) explains approximately 54% (p<0.01) of the variation in change in BMI. However, for exposure on the weekends, LASSO, at the minimum Mallows C_p_ [20], had all coefficients equal to zero, and consequently, selected no variables. This means that exposure factors (numbers of close friends or numbers of acquaintances with specific behavior and health-related outcomes) on weekends could not explain variation in the change in BMI.

Overall, a comparison of the models based on duration of interactions, in Table 1 to the models based on numbers of subjects exposed to, in Table 2, shows that models based on duration of interactions (see Table 1) have the better R^2^ and AIC values. Therefore, models based on duration of interactions explain variation in change in BMI better than the models on the numbers of subjects exposed to. The optimal model is a model based on duration of interactions aggregated for the entire period of the study. This model indicates that the entire period of interaction is more important than subsets of itself (weekdays and weekends) in explaining change in BMI.

## Discussion

Change in BMI showed a strong association with social exposure to acquaintances who gained weight. However social exposure to close friends who experienced a weight change did not show any significant correlation with change in BMI, which is different from the result by Christakis and Fowler [8] that suggested that there is a strong correlation between the obesity status of an individual and that of the individual's friend. This result by Christakis and Fowler [8] may be the consequence of a sampling procedure that is biased towards selecting only friends and relatives. Social exposure to close friends that are not stressed is the only close friends based social exposure that appeared correlated with change in BMI. This further highlights the significance of social exposure to acquaintances.

In the model, exposure to acquaintances that gained weight has a negative relationship with change in BMI whereas exposure to acquaintances that are not stressed shows a positive relationship with change in BMI. In the western, world slenderness is associated with happiness, success, youthfulness, and social acceptability [23]. Therefore the beneficial effect of social exposure to acquaintances who gained weight may be due to the increased salience of weight gain, which can help individuals' motivation to take preventative action. A similar mechanism may be behind the success of team-based weight loss programs and participatory TV shows such as the “Biggest Loser” where obese and overweight contestants battle to lose the most weight.

Interestingly, social exposure alone is more important in explaining variation in change in BMI than personal choices, since exposure explains more than half of the 68% of change in BMI within the optimal model, which is a combination of exposure and personal choices; and the personal attributes (gender) and health related choices, healthy diet category and ability to manage stress alone do not explain the change in BMI. This finding emphasizes the importance of holistic and accurate measures of social exposure. It shows that social interactions, which are overlooked in obesity and weight studies, are particularly useful in explaining change in BMI.

Personal attribute and gender were included in the model, based on studies that indicate BMI is not independent of gender [24], [25] and suggests that healthy diet [26], [27] and stress [28], [29] are related to weight and weight change. However, the lack of a wide range in ages and small population size limits the generality of the study although the study population includes subjects from different races that are with varying levels of income, and from different cultures. This model does not take into account mobility changes of individuals into and out-of communities and therefore it is not clear the importance of mobility in this model. Future research shall asses this model in larger and more diverse communities to understand the generalizability of the model.

## Conclusion

To our knowledge this is the first study that integrates both interactions with close friends and acquaintances (using detailed technically advanced methods) in combination with individual health-related information to explain change in BMI. We predict change in BMI by social exposure to people in a social network, both close friends and acquaintances, thereby eliminating any bias that may result from sampling only close friends or acquaintances. This study suggests that exposure is more important than individual choices in explaining change in weight, and highlights the importance of people that an individual is exposed to in their community. These findings further suggest that research work on obesity and weight change-related issues need to take cognizance of social exposure especially to acquaintances, since it may be as critical if not more important than the exposure to close friends. The ability to measure social interaction using embedded sensing techniques in the real world presents a new avenue to understand individual behavior and to achieve the ultimate goal of Public Health - guaranteeing every individual in the community a standard of living adequate for the maintenance of health.
